# The Students Guide to the Practice of Midwifery

**Published:** 1876-08

**Authors:** 


*The Students' Guide to the Practice of Midwifery.* By D. Lloyd Roberts, M. D., M. R. C. P., Lond. Philadelphia : Lindsay & Blakiston, 1876. Buffalo : T. Butler & Son.

This work is a manual written for the instruction of students in one of the most important branches in the practice of medicine. It is intended to impart to the young student that practical knowledge which he will need in his professional life. The work opens with a brief sketch of the anatomy of the parts concerned in parturition, following which the physiology of generation is considered. A concise account of the mechanism of labor is given, without a knowledge of which the subject can not be properly understood. The succeeding chapters treat in order placenta previa, signs of pregnancy, diseases of pregnancy, abortion, diseases of the membranes, super fœtation, extra uterine pregnancy, stages of labor, management of natural labor, the puerperal state, obstructed labor, protracted and hasty parturition, accidental hemorrhage, rupture of the uterus, induction of premature labor, version, the forceps, craniotomy cæsarean section, puerperal convulsions, puerperal mania, phlegmasia alba dolens, puerperal fever, thrombosis and embolism, pelvic cellulitis and pelvic peritonitis. The work is illustrated in a suitable manner, the drawings all being well calculated to render the author's meaning more easily comprehended. The discussion of the merely theoretical part of obstetrics is wisely left out of the work, as they would certainly not be suitable to a book of its pretentions, and would only serve to befog the mind of the student.

As a hand book it will be of value to students, and may well occupy the time of practitioners in a careful perusal. Dr. Roberts is an easy writer and evidently speaks largely from personal experience.

